# *Maytenus disticha* Extract and an Isolated β-Dihydroagarofuran Induce Mitochondrial Depolarization and Apoptosis in Human Cancer Cells by Increasing Mitochondrial Reactive Oxygen Species

**DOI:** 10.3390/biom10030377

**Published:** 2020-02-29

**Authors:** Iván González-Chavarría, Felix Duprat, Francisco J. Roa, Nery Jara, Jorge R. Toledo, Felipe Miranda, José Becerra, Alejandro Inostroza, Alexandra Kelling, Uwe Schilde, Matthias Heydenreich, Cristian Paz

**Affiliations:** 1 Departamento de Fisiopatología, Facultad de Ciencias Biológicas Universidad de Concepción, Concepción 4030000, Chile; ivangonzalez@udec.cl (I.G.-C.); felixdavid.duprat@gmail.com (F.D.); fraroa@udec.cl (F.J.R.); jotoledo@udec.cl (J.R.T.); 2 Departamento de Farmacología, Facultad de Ciencias Biológicas Universidad de Concepción, Concepción 4030000, Chile; neryalejara@udec.cl; 3 Laboratorio de Productos Naturales y Descubrimiento de Fármacos, Departamento de Ciencias Básicas, Universidad de La Frontera, Temuco 4780000, Chile; alvaro.miranda@ufrontera.cl (F.M.); jbecerra@udec.cl (J.B.); 4 Departamento de Botánica, Facultad de Ciencias Naturales y Oceanográficas, Universidad de Concepción, Concepción 4030000, Chile; alejandro.inosm@gmail.com; 5 Universität Potsdam, Institut für Chemie, Karl-Liebknecht-Str. 24-25, D-14476 Potsdam, Germany; kelling@chem.uni-potsdam.de (A.K.); uschilde@uni-potsdam.de (U.S.); mheydenr@uni-potsdam.de (M.H.)

**Keywords:** *Maytenus disticha*, *β*-dihydroagarofuran-type sesquiterpene, dihydromyricetin, dihydromyricetin-3-O-*β*-glucoside, cytotoxic activity, Mitochondrial ROS

## Abstract

*Maytenus disticha* (Hook F.), belonging to the Celastraceae family, is an evergreen shrub, native of the central southern mountains of Chile. Previous studies demonstrated that the total extract of *M. disticha* (MD) has an acetylcholinesterase inhibitory activity along with growth regulatory and insecticidal activities. β-Dihydroagarofurans sesquiterpenes are the most active components in the plant. However, its activity in cancer has not been analyzed yet. Here, we demonstrate that MD has a cytotoxic activity on breast (MCF-7), lung (PC9), and prostate (C4-2B) human cancer cells with an IC_50_ (µg/mL) of 40, 4.7, and 5 µg/mL, respectively, an increasing Bax/Bcl2 ratio, and inducing a mitochondrial membrane depolarization. The β-dihydroagarofuran-type sesquiterpene (MD-6), dihydromyricetin (MD-9), and dihydromyricetin-3-O-β-glucoside (MD-10) were isolated as the major compounds from MD extracts. From these compounds, only MD-6 showed cytotoxic activity on MCF-7, PC9, and C4-2B with an IC_50_ of 31.02, 17.58, and 42.19 µM, respectively. Furthermore, the MD-6 increases cell ROS generation, and MD and MD-6 induce a mitochondrial superoxide generation and apoptosis on MCF-7, PC9, and C4-2B, which suggests that the cytotoxic effect of MD is mediated in part by the β-dihydroagarofuran-type that induces apoptosis by a mitochondrial dysfunction.

## 1. Introduction

Cancer is one of the leading causes of morbidity and mortality worldwide. Lung, breast, and prostate cancer represent more than 32% of total cancer [1]. The drugs used in cancer treatment aim to reduce proliferation, promote the apoptosis, reduce the angiogenesis, or prevent the invasion and metastasis [2]. However, cancer chemotherapies show a series of side effects and a high percentage of patients develop drug resistance, leading to a decrease in their life expectancy [3]. Thus, the discovery of new drugs against cancer is an important challenge. In this sense, chemical compounds from plants are an important source for new drug developments against cancer [4,5,6]. Vinca alkaloids and taxanes are a good example of drugs derived from plants or fungus that are currently used in cancer treatment [7,8,9].

*Maytenus disticha* is an evergreen shrub, native of the central southern mountains of Chile, which belongs to the Celastraceae family. This family has approximately 88 genera and 1300 species of plants. In Chile, the *Maytenus* genus includes four endemic species: *Maytenus boaria* Mol., *Maytenus disticha* (Hook. F.) Urban, *Maytenus magellanica* (Lam.) Hook. F., and *Maytenus chubutensis* (Speg.) Lourteig. O’Don. & Sleumer. Plants belonging to the Celastraceae family produce characteristic dihydro-*β*-agarofuran type sesquiterpenes, which have been used as taxonomic markers for the family [10]. A broad range of activities of the *Maytenus* genus have been described, showing, for example, inhibitory effects on acetylcholinesterase [11], antifungal activity [12], and anti-proliferative properties [13,14]. *Maytenus procumbens* exhibited anti-proliferative effects and induced apoptosis in human cervical cancer cells (HeLa) [15]. Interestingly, some dihydroagarofuran-type sesquiterpenes, from plants of the *Maytenus* genus, showed an insecticidal and trypanocidal activity by producing mitochondrial dysfunction and ROS generation [16,17]. In this sense, mitochondrial dysfunction and elevated oxidative stress have been described as differentiating characteristics of cancer cells compared to normal cells [18]. Therefore, molecules with the ability to increase mitochondrial ROS over a critical threshold for producing apoptosis, are promising therapeutic agents in cancer. An interesting molecule is the natural compound gracillin, which induces apoptosis in human cancer cell lines, suppresses the growth of xenograft tumors in mice, and decreases mitochondria-mediated cellular bioenergetics by producing ROS [19]. A number of other molecules showing anti-cancer effects by targeting mitochondrial or intracellular ROS production have been studied [20,21].

In the present research, we hypothesize that the total extract of *Maytenus disticha* and the compounds isolated from this plant have cytotoxic activity against prostate, breast, and lung cancer cells through the induction of a ROS-mediated mitochondrial dysfunction.

## 2. Materials and Methods

NMR spectra were recorded on a Bruker AVANCE III 600 MHz spectrometer (^1^H: 600 MHz, ^13^C: 150 MHz, Bruker Biospin GmbH, Rheinstetten, Germany), and TMS were used as internal standard. HRESIMS were obtained using on DSQII GC/MS-system (Axel Semrau GmbH & Co., Sprockhövel, Germany). FTIR spectra were measured on a Nicolet 6700 from Thermo Electron Corporation with the ATR-unit Smart Performer (Thermo Electron Corporation, Waltham, MA, USA). Melting points were determined on a Melting Point SMP10 (Cole-Parmer, Staffordshire, UK), they are uncorrected. Preparative chromatography was performed using Merck silica gel 60 and Sephadex LH-20 (25−100 μm; Aldrich, Santiago, Chile). Fractions were concentrated in a rotavap Büchi model R100. Analytical chromatoplates GF254 were purchased from Merck Co. (Santiago, Chile). Solvents used in this study were distilled prior to use and dried over appropriate drying agents. The X-ray crystallography analysis was performed with crystal embedded in perfluoropolyalkylether oil and mounted within a MicroGripper (Jena Bioscience, Jena, Germany). The data collection was performed at 210 K on a STOE StadiVari diffractometer equipped with a four-circle goniometer (open Eulerian cradle), a Genix Microfocus X-ray source (Mo) with a graded multilayer mirror, and a Pilatus 200 K detector (5210 frames, 5 s exposure time per frame, Δ*ω *= 0.5°; detector distance 60 mm (Dectris, Baden-Daetwil, Switzerland). The data were corrected for Lorentz and polarization effects using the software X-Area. The structures were solved by direct methods using SHELXS-2013/2 [22] and refined by full-matrix least squares on *F*^2^ using the program SHELXL-2014/7 [23]. All non-hydrogen atoms were refined anisotropically. The hydrogen atom of the O-H group was located from the difference Fourier maps and freely refined. The other hydrogen atoms were calculated in their expected positions and refines using a riding model with C-H = 0.99 Å (-CH), C-H = 0.98 Å (-CH_2_), and C-H = 0.97 Å (-CH_3_), as well as *U*_iso_(H) = 1.2*U*_eq_(CH_,_ CH_2_) and *U*_iso_(H) = 1.5*U*_eq_(CH_3_). The absolute configuration was determined by an anomalous dispersion effect (FLACK parameter 0.03(14)) [24]. For the visualization, the programs ORTEP-3 for windows [25] and DIAMOND [26] were used. CCDC-1958157 contains the supplementary crystallographic data for this paper. These data can be obtained free of charge from The Cambridge Crystallographic Data Centre via www.ccdc.cam.ac.uk/data_request/cif.

### 2.1. Plant Material

**Figure 1:**
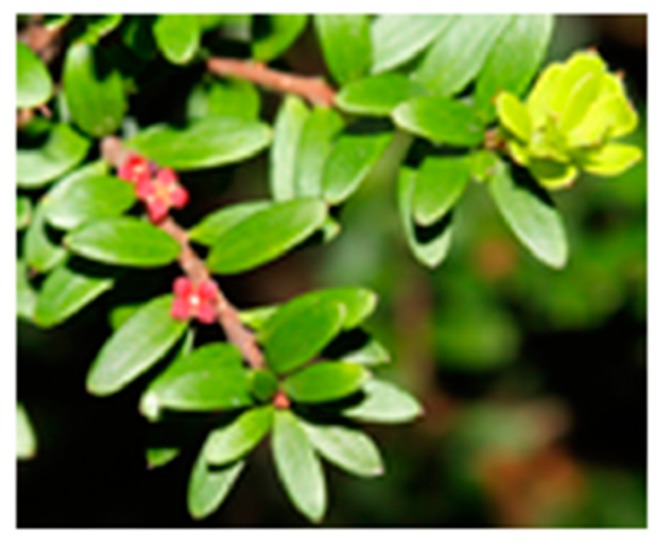
Photography of the *Maythenus disticha* shrub.

Aerial parts of *Maytenus disticha* were collected in Pucón, IX Region of Chile, in November 2017, at the end of the spring season (Figure 1). A voucher specimen was deposited at the herbal collection of the laboratory of natural products (C. Paz) Universidad de La Frontera, Chile.

### 2.2. Extraction of Maytenus disticha

A total of 7.0 kg of aerial parts of *Maytenus disticha* (humid weight) were dried, powdered, and extracted by maceration with ethyl acetate/methanol (1:1 v/v) for 3 days (10 L × 2-fold). The organic solvent was evaporated in vacuo giving a crude extract: 950 g, gummy oil. This extract was further fractionated by silica gel column chromatography, giving a primary fractioning of ten fractions (*Maytenus disticha* (MD)-F1 to MD-F10) by using an increasing polarity of solvents from n-hexane to EtOAc. Fractions MD-F1 to MD-F4 displayed carotenoids, unsaturated fatty acids, and β-sitosterol, followed by a concentrate of chlorophyll in the MD-F5 fraction. MD-F6 showed a single spot by TLC, which was purified by CC with n-hexane/EtOAc (3:2 v/v) giving MD-6 (70 mg, colorless crystals, 0.001% yield), whereas the purification of MD-F9 with n-hexane/EtOAc (1:4 v/v) gave MD-9 (150 mg, white solid, 0.0021% yield). A subsequent purification of MD-F10 by CC with EtOAc gave MD-10 (480 mg, white solid, 0.0069% yield).

**Scheme 1:**
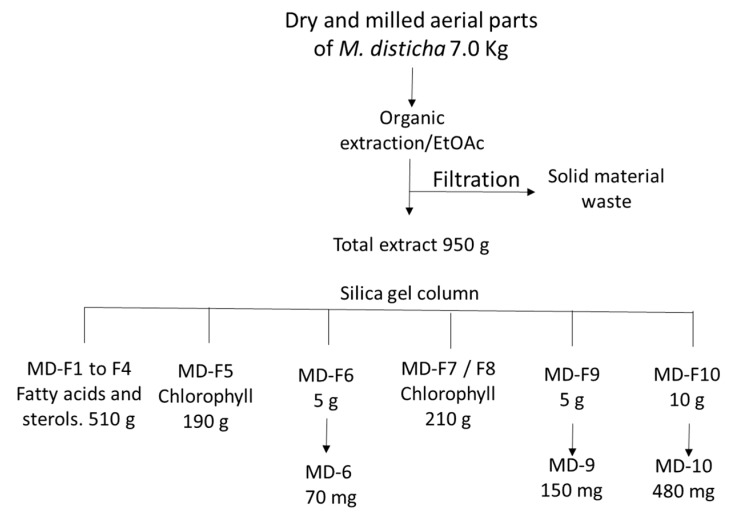
Method of obtaining extracts, fractions, and compounds from aerial parts of *Maytenus disticha*.

All compounds were recrystallized by slow evaporation at 20 °C from MeOH/EtOAc (1:1 v/v) to obtain crystals or amorphous solids with over 99% purity by NMR. Pure compounds and fractions were air-dried and frozen at −20 °C until use; procedure resumed in Scheme 1.

### 2.3. Cell Culture

Prostate cancer (C4-2B), breast cancer (MCF-7), and lung cancer (PC9) cells were obtained from the American Type Culture Collection and were grown in a RPMI medium with FBS 10% (v/v) at 37 °C in a humidified atmosphere containing 5% CO_2_.

### 2.4. Cytotoxic Assay and IC_50_ Determination

MD extract, MD-6, MD-9, and MD-10 were dissolved in DMSO and stored at −20 °C. The solution was diluted with DMSO to obtain working concentrations. The IC_50_ determination of the MD extract and the isolated compounds was analyzed by clonogenic assay, the cells were grown at a density of 1.000 per well in a six wells plate and then the cells were incubated with an increased concentration of the MD extract (0–500 µg/mL) or the isolated compounds (0–100 µM) in RPMI media with a DMSO concentration minor to 1% (v/v). After 24 h, the extract was removed and the cells were grown for 14 days with refreshed RPMI media. The colonies were stained with crystal violet, images were photo-documented with LI-COR ODYSSEY CLX, and the total colony numbers were counted for each condition.

### 2.5. Mitochondrial Superoxide and Celllular ROS Determination

A total of 20.000 prostate cancer (C4-2B), breast cancer (MCF-7), and lung cancer (PC9) cells were seeded in 96 well plates for fluorescence. Cells were pre-incubated with 5 µM MitoSox Red (Invitrogen, Waltham, MA, USA) for Mitochondrial superoxide or 5 µM CM-H*_2_*DCFDA for general oxidative stress indicator (Invitrogene) for 15 min and treated for 6 h with 100 µg/mL MD, 50 µM MD-6, or 50 µM carbonyl cyanide-4-(trifluoromethoxy)phenylhydrazone, FCCP (positive control). The fluorescence for both probes was analized using the Synergy HTX multi-mode reader (Biotek, Winooski, VT, United States). MitoSox Red probe was also analyzed by epifluorescence microscopy (OLYMPUS IX81, Shinjuku City, Tokyo, Japan).

### 2.6. Mitochondrial Membrane Potential Analysis

Since mitochondrial membrane depolarization is induced during an apoptosis process, the mitochondrial membrane potential (Δψ) was analyzed using the probe JC1 (BD Biosciences, San Jose, CA, USA). MCF-7, C4-2B, and PC9 cells were treated with 100 µg/mL MD or carrier for 24 h. Then, the cells were incubated with 5 µM JC1 for 15 min, washed and maintained with a RPMI medium. The mitochondrial membrane potential was analyzed by epifluorescence microscopy (OLYMPUS IX81) at 530 and 590 nm. At high Δψ, JC-1 forms aggregates producing red fluorescence; at low Δψ, JC-1 forms monomers producing green fluorescence. Therefore, a decrease in red fluorescence is indicative of mitochondrial membrane depolarization.

### 2.7. Real-Time PCR for Pro-Apoptotic and Anti-Apoptotic Gene Expression Analysis

The apoptosis signature was evaluated by real-time PCR in cells treated with MD 100 µg/mL for 24 h. Total RNA was extracted using Trizol reagent (Invitrogene, USA). Real-time PCR for BAX (forward 5′-GCCAGCAAACTGGTGCTCAA-3’, reverse 5´-CCAACCACCCTGGTCTTGGA-3´) and BCL-2 (forward 5´-TGATGGGATCGTTGCCTTATGC-3´, reverse 5´-ACCAATTTCCTGTGCGAGACTT-3´) was performed using the KAPA SYBR^®^ FAST qPCR KIT (KAPA Biosystems, Woburn, MA, USA) and Stratagene MX3000P equipment. The results were analyzed as 2^−*ΔΔ*CT^ relative quantification. The comparative threshold cycle values were normalized for *β*-actin mRNA.

### 2.8. Detection of Apoptosis by Flow Cytometry Using Annexin V/PI Assay

The MCF-7, PC-9, and C4-2B cells were treated with MD (100 μg/mL) or MD-6 (50 μM) or DMSO for 24 h. Then, the cells were stained with the Alexa Fluor® 488 Annexin V/Dead Cell Apoptosis Kit (Thermo Fisher Scientific, Waltham, MA, USA) as indicated by the supplier. The acquisition was on a BD FACSAria III flow cytometer (BD Biosciences) with a 488 nm argon ion laser and a 635 nm red diode laser. The light scatter and fluorescent parameters were set at logarithmic gain, and cells were identified according to their characteristic forward and side scatter properties, with 20,000 total events per sample acquired. Flow cytometric analysis was conducted by FlowJo Software v10.6.

### 2.9. DAPI Staining to Visualize Apoptosis

To assess apoptosis after drug treatment, cells were stained with 4’,6-diamidino-2-phenylindole (DAPI) to detect nuclear condensation and fragmentation. The cells were treated with MD (100 μg/mL) or MD-6 (50 μM) or DMSO for 24 h. Then, the cell culture medium was discarded, and the cells were permeabilizated with 0.1% (v/v) of triton ×100, washed, and incubated with 1 μg/mL of DAPI solution (Thermo fisher scientific). The cells were washed with PBS and examined by epifluorescence microscopy (OLYMPUS IX81).

### 2.10. Statistical Analyses

Data are presented as means +/− standard deviation (SD). The statistical analysis was performed with the GraphPad Prism 7.0 software. Multiple comparisons were analyzed by one-way ANOVA with a Dunnett post-test, and a *p* value of < 0.05 was considered to be significant.

## 3. Results

Looking for natural anticancer agents, we previously studied the Chilean native tree *Maytenus boaria,* isolating four dihydro-*β*-agarofurans sesquiterpenoids from organic extracts of its seeds [27,28]. However, these compounds were not active in cancer cells models, specifically against MCF-7, PC9, and C4-2B cells (data not shown). Thereafter, attention was paid to another member of this genus, *Maytenus disticha*, which grow near the central southern mountains of Chile, gathering 7.0 kg of aerial parts. This material was extracted by maceration with ethyl acetate/methanol 1:1, giving 950 g of total extract.

### 3.1. Chemical Characterization of Compounds Purified from MD Extract

**Figure 2:**
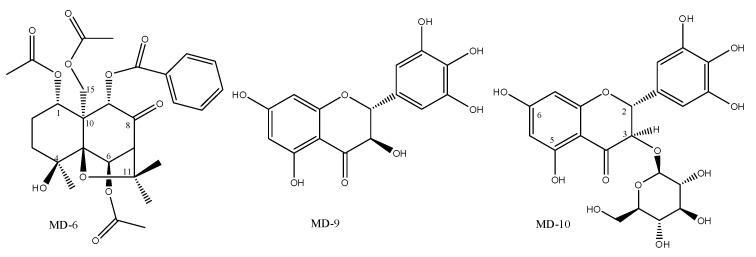
Molecular structures of pure compounds obtained from *Maytenus disticha*. *Maytenus disticha* (MD)-6: (5S,5aS,6S,9S,9aS,10R)-5a-(Acetoxymethyl)-5-(benzoyloxy)-9-hydroxy-2,2,9-trimethyl-4 oxooctahydro-2H-3,9a-methanobenzo[b]oxepine-6,10-diyl diacetate. MD-9: Ampelopsin, dihydromyricetin. MD-10: Dihydromyricetin-3-glucosid.

The purification of the extract of the aerial parts of *Maytenus disticha* by column chromatography, determined three natural compounds as main secondary metabolites (Figure 2). Their structures were determinate using 1D and 2D NMR (assignments are summarized in Table S1). Melting points were measured for MD-6, MD-9, and MD-10 and results showed a melting point of 236 °C, 215 °C, and 202 °C, respectively, for each molecule.

### 3.2. X-Ray Structure Analysis of MD-6

**Figure 3:**
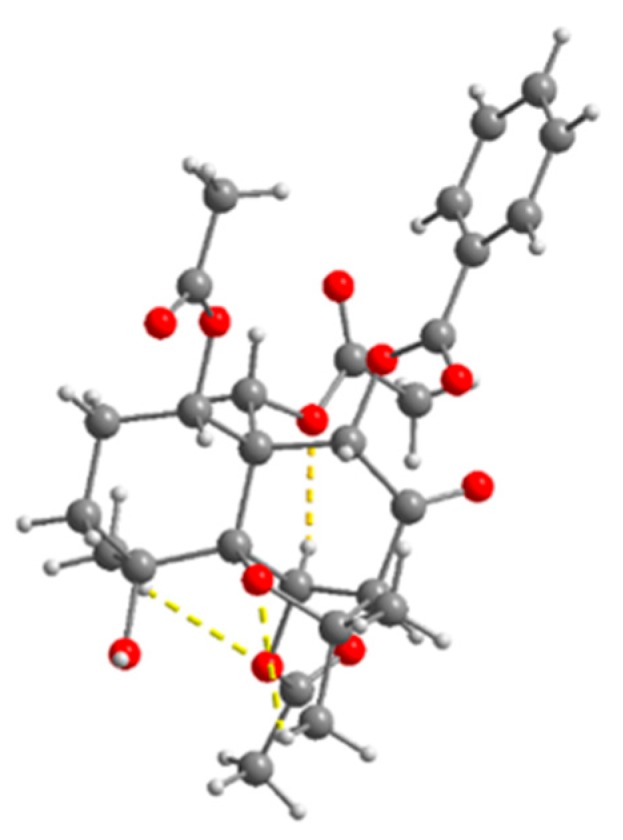
Molecular structure of MD-6. Hydrogen bonds are shown as yellow dashed lines.

All compounds were recrystallized by slow evaporation at 20 °C from MeOH/EtOAc 1:1 v/v. However, only MD-6 gave suitable crystals for X-Ray diffraction, meanwhile MD-9 and MD-10 were obtained as amorphous solids. The structure of MD-6 was unambiguously determined by single-crystal X-ray structure analysis (Figure 3). The absolute configuration could be estimated by effect of anomalous dispersion and in agreement with previous compounds [29]. Atomic labeling is shown in Figure S1. Crystal Data, Details of Intensity Measurements, and Structure Refinement are given in Table S2. For the Packing diagram, refer to Figure S2. Hydrogen-bonding parameters are shown in Table S3. Selected bond lengths and bond angles are displayed in Tables S4 and S5, respectively. The compound is a tricyclic dihydro-β-agarofuran sesquiterpenoid. The molecular structure consists of a ten-membered decaline ring with hydroxyl, oxo, benzoyloxy, and three acetoxy substituents. The ring is fused with a tetrahydrofurane ring between C5 and C7, which has two methyl groups at C11. The structure is closely related to that of Bilicularin A and Bilicularin B [30]. In contrast to MD-6, the latter two compounds do not contain a hydroxyl group in position 4. Bilicularin A has a secondary hydroxy group in position 8, while Bilicularin B is the oxidation product, i. e., it contains the carbonyl group in that position, as is the case in MD-6. The hydroxy group at C4 is bisectionally positioned and the oxo group at C8 is equatorial with respect to their cyclohexane rings. The structure is stabilized by intramolecular non-classical C-H···O hydrogen bonds. Furthermore, intermolecular C-H···O as well as O-H···O hydrogen bonds can be observed (Figure S2). The unit cell contains no residual solvent accessible void.

### 3.3. MD Total Extracts Promote Cytotoxicity on Human Cancer Cells

**Figure 4:**
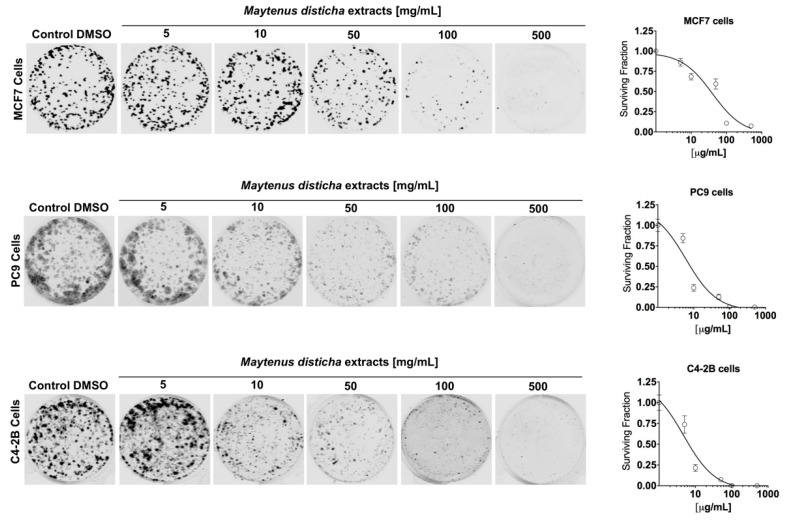
*Maytenus disticha* extract has cytotoxic effects in human cancer cells. The cytotoxicity was evaluated by clonogenic assay in MCF-7, PC9, and C4-2B cells treated with 5–500 µg/mL of MD extract for 24 h. The IC_50_ were calculated using the GraphPad prism software for each cell line.

The cytotoxic effect of the MD extract was evaluated on MCF-7, PC9, and C4-2B cells by clonogenic assays. The results showed that the MD extract significantly reduced the cell viability on MCF7 cells by 14, 32, 41, 90, and 93% at concentrations of 5, 10, 50, 100, and 500 µg/mL, respectively. At the same concentrations, on PC9 cells, the MD extract reduced the cell viability by 16, 77, 88, 99, and 99%, respectively, while on C4-2B cells, the extract reduced the cell viability by 27, 80, 90, 99, and 99%, respectively. Thus, the IC_50_ for the MD extract was 40, 4.7, and 5.0 µg/mL for the MCF7, PC9, and C4-2B cells (Figure 4).

### 3.4. MD Total Extracts Increase the BAX/BCL-2 Ratio in Prostate, Breast, and Lung Cancer Cells

**Figure 5:**
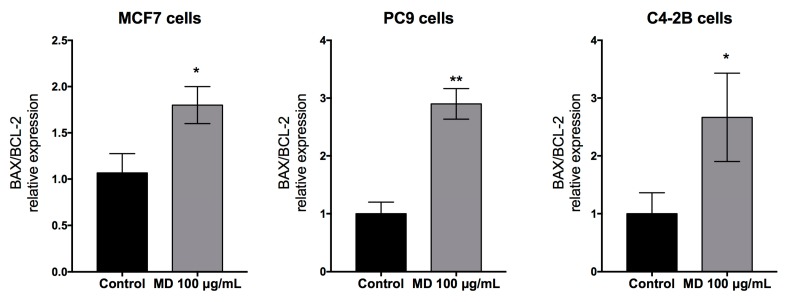
*Maytenus disticha* extracts increase the BAX/BCL2 ratio in breast, lung, and prostate cancer cells. MCF-7, PC9, and C4-2B cells were treated with 100 µg/mL of MD extract for 24 h and the expression of BAX and BCL2 were analyzed by real-time PCR. The data represent the means ± S.D. of three independent experiments performed in triplicate, and statistically analyzed using one-way analysis of variance and Dunnett’s post-test; (** *p* ≤ 0.01, * *p* ≤ 0.05).

The programmed process cell death, or apoptosis, is generally characterized by distinct morphological characteristics, energy-dependent biochemical mechanisms, and a transcriptional signature imbalance that favors the expression of pro-apoptotic proteins, such as BAX, in detriment of anti-apoptotic proteins, such as BCL-2. We analyzed the expression rate of BAX/BCL-2 in MCF-7, C4-2B, and PC9 cells treated with MD extracts (100 µg/mL) for 24 h using real-time PCR. The results showed that MD extract significantly increased the BAX/BCL-2 ratio by 1.7-, 2.7-, and 2.5-fold in MCF-7, PC9, and C4-2B cells, respectively (Figure 5).

### 3.5. MD Total Extract Promotes a Mitochondrial Membrane Potential Depolarization

**Figure 6:**
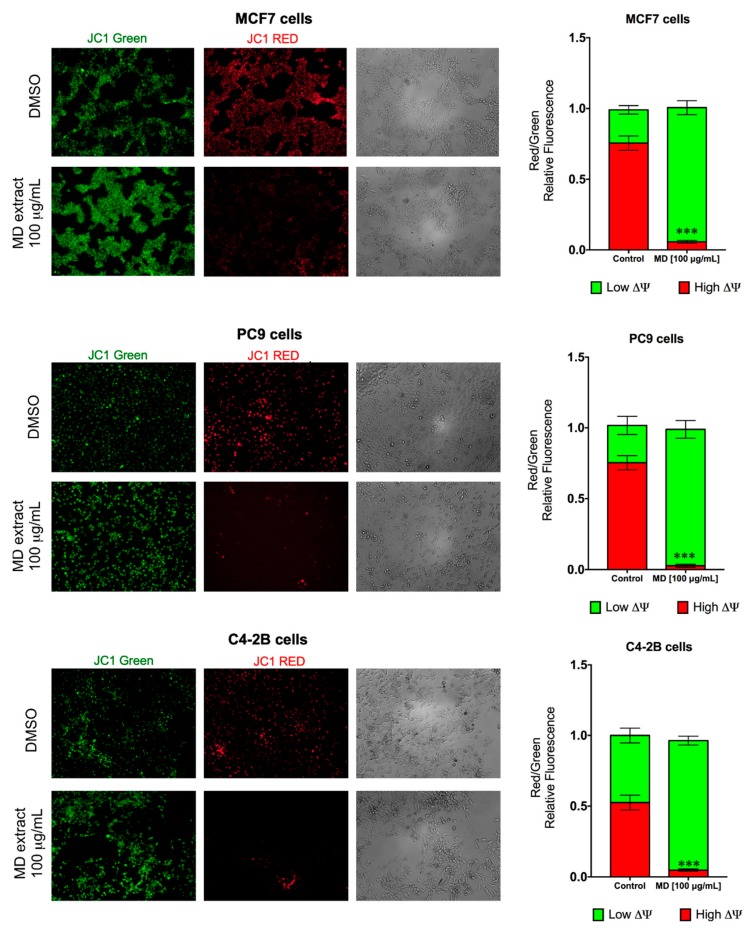
*Maytenus disticha* extract promotes a mitochondrial membrane potential depolarization. The mitochondrial membrane potential **Δψ** was evaluated using JC1 assays and epifluorescence microscopy in MCF-7, PC9, and C4-2B cells treated with MD extract (100 µg/mL) for 24 h. The red color is associated with a high Δψ and the green color with a low Δψ. The data represent the means ± S.D. of three independent experiments performed in triplicate, and statistically analyzed using t student test; (*** *p* ≤ 0.001). The microphotographs were acquired with an Olympus IX81 DSU microscope (20× magnification).

One of the effects of an apoptosis process is the generation of changes in the mitochondrial membrane potential promoting its depolarization. Based on this background, we evaluated the mitochondrial membrane potential (**Δψ**) in MCF-7, C4-2B, and PC9 cells treated with MD extract (100 µg/mL) for 24 h using JC1 assays and epifluorescence microscopy. The result showed that the MD extract decreased the mitochondrial membrane potential **Δψ** by 92%, 90%, and 96% for MCF-7, C4-2B, and PC9, respectively, compared to the control. These results corroborate that the increment in the BAX/BCL2 ratio induced by MD has a functional repercussion inducing the apoptosis. (Figure 6).

### 3.6. The β-Dihydroagarofuran-Type Sesquiterpene, MD-6, Promotes Cytotoxicity on Human Cancer Cells

**Figure 7:**
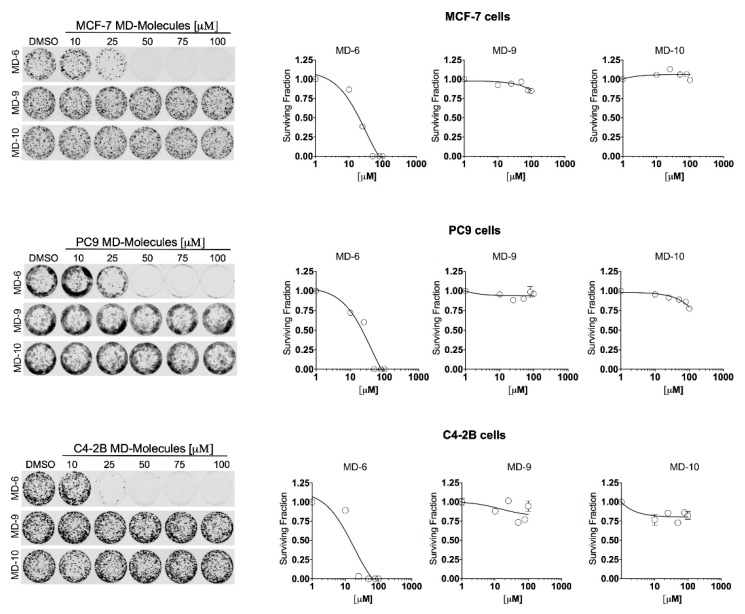
Cytotoxic effects of MD-6, MD-9, and MD-10 on human cancer cells. The cytotoxicity of isolated molecules were evaluated by clonogenic assay on MCF-7, PC9, and C4-2B cells treated with increased concentrations of MD-6, MD-9, and MD-10 for 24 h.

To find some molecules that promote apoptosis in total extract, the cytotoxic effects of isolated molecules from *Maytenus disticha* (MD-6, MD-9, and MD-10) were evaluated on MCF-7, C4-2B, and PC9 cells by clonogenic assay. The results showed that the *β*-dihydroagarofuran-type sesquiterpene (MD-6) has a cytotoxic effect on MCF-7, PC9, and C4-2B cells with an IC_50_ of 42.19, 31.02, and 17.58 µM, respectively. However, no cytotoxic effect was observed for dihydromyricetin (MD-9) and dihydromyricetin-3-O-*β*-glucoside (MD-10) on any tested cell (Figure 7).

### 3.7. MD and MD-6 Induce Mitochondrial Oxidative Stress in Prostate, Breast, and Lung Cancer Cells

**Figure 8:**
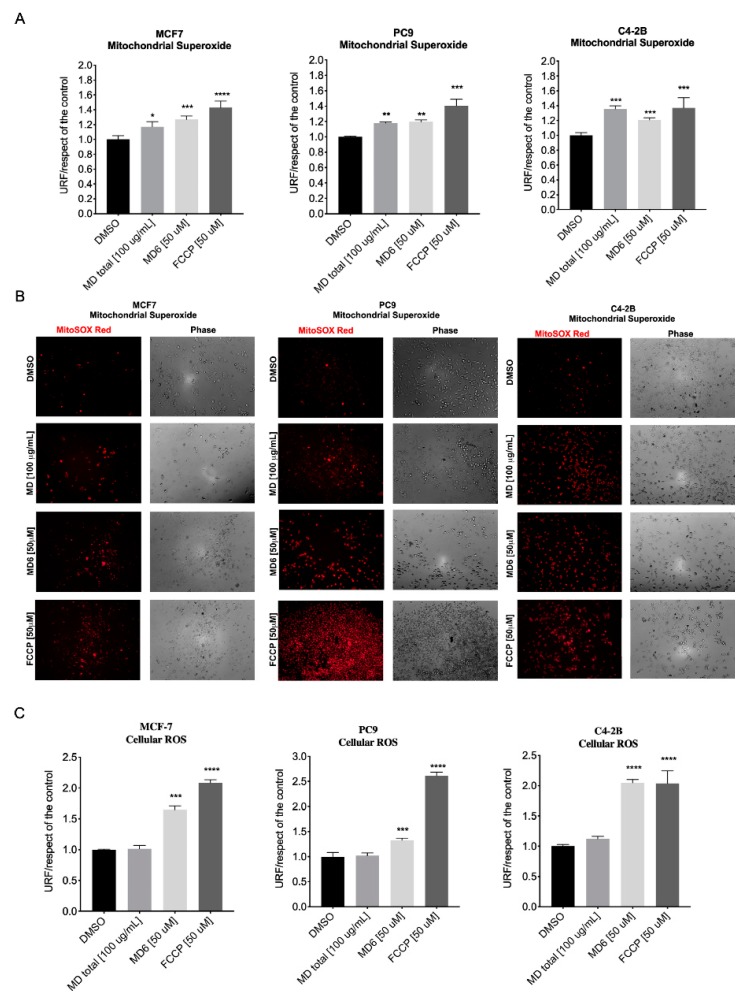
Effect of MD and MD-6 on mitochondrial and cell ROS generation in human cancer cells. MCF-7, PC9, and C4-2B cells were treated with 100 µg/mL MD extract, 50 µM MD-6, or 50 µM FCCP for 6 h. Mitochondrial superoxide and cell ROS levels were analyzed using MitoSOX Red and CM-H2DCFDA probes, respectively. (**A**) Mitochondrial superoxide determination by fluorimetry, (**B**) epifluorescence microscopy, the microphotographs were acquired with an Olympus IX81 DSU microscope (20× magnification). (**C**) Cell ROS determination by fluorimetry. The data represent the means ± S.D. of three independent experiments performed in triplicate, and statistically analyzed using one-way analysis of variance and Dunnett’s post-test; (**** *p* ≤ 0.0001, *** *p* ≤ 0.001, ** *p* ≤ 0.01, * *p* ≤ 0.05).

A previous report showed that a dihydroagarofuran-type sesquiterpene presented insecticidal and trypanocidal activity, which is mediated by mitochondrial dysfunction and ROS generation. Therefore, we evaluate whether the MD-induced apoptosis observed in cancer cells is related to mitochondrial and cell oxidative stress. Using the probe MitoSOX Red, we analyzed the mitochondrial superoxide production in MCF-7, PC9, and C4-2B cells treated with MD total extract (100 µg/mL) and MD-6 (50 µM). The results showed that in both conditions, the levels of mitochondrial superoxide increased compared to the control cells, in the three cell lines analyzed (Figure 8). This effect was proved by fluorometric assays (Figure 8A) and fluorescence microscopy experiments (Figure 8B), using FCCP as a positive control for the production of mitochondrial ROS. In addition, we analyzed the general ROS production in treated MCF-7, PC9, and C4-2B cells, using the probe CM-H_2_DCFDA. Only MD-6, but not the MD total extract, induced an increase in the cell ROS levels compared to control cells in the three cell lines analyzed (Figure 8C).

### 3.8. MD and MD-6 Induce Apoptosis in Prostate, Breast, and Lung Cancer Cells

**Figure 9:**
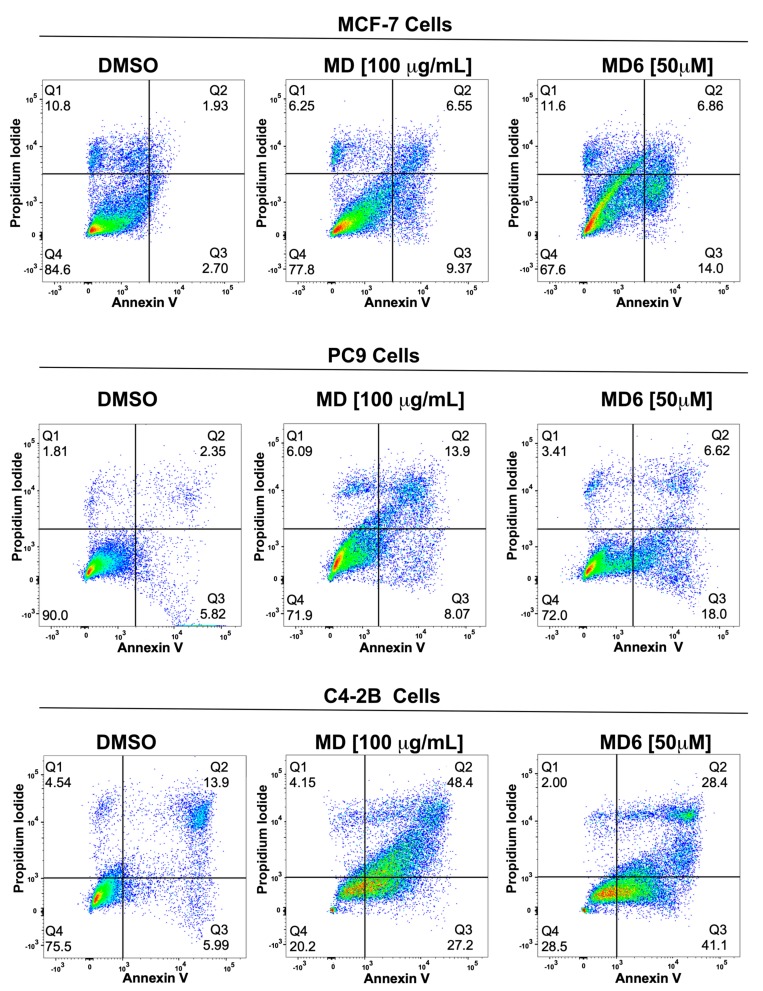
Apoptosis analysis on breast, lung, and prostate cancer cells treated with MD and MD-6 for 24 h. Detection of apoptosis by flow cytometry using annexin V/PI Assay.

**Figure 10:**
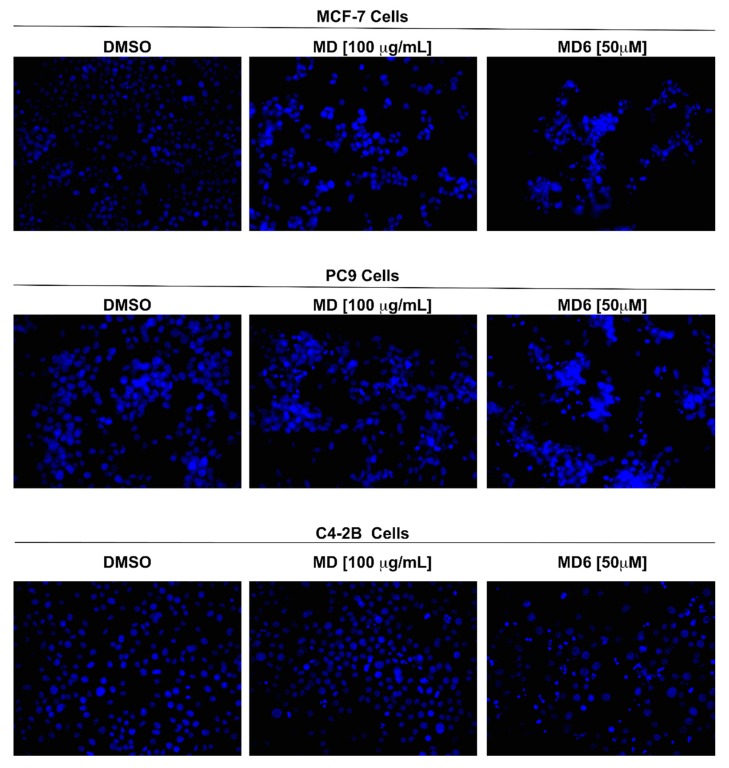
Apoptosis analysis on breast, lung, and prostate cancer cells treated with MD and MD-6 for 24 h. Detection of apoptosis by microscopy using DAPI stain. The microphotographs were acquired with an Olympus IX81 DSU microscope (20× magnification).

Apoptosis was analyzed by Flow Cytometry using AnnexinV/PI Assay and nuclear-stained with DAPI for microscopy in MCF-7, PC-9, and C4-2B cells treated with MD (100 μg/mL) or MD-6 (50 μM) or DMSO for 24 h. For Flow Cytometry the combined Annexin V and PI reactivity allowed the classification of cells into four groups: early apoptotic cells Annexin V (+) and PI (−), late apoptotic or dead cells (Annexin V (+) and PI (+)), dead cells Annexin V (−) and PI (+), and live cells Annexin V (−) and PI (−). The treatment of MCF-7, PC-9, and C4-2B cells with MD (100 μg/mL) or MD-6 (50 μM) decreased the percentage of live cells and induced high affinity for Annexin V, as shown by the right shift of the scatter plot compared with that of DMSO treated cells, indicating early apoptosis (Figure 9). Furthermore, the MD and MD-6 increased the number of cells positive for Annexin V (+) and PI (+), which denotes that a late apoptosis occurs. MD-6 (50 μM) showed more activity to induce early apoptosis compared to MD on all cell lines assayed. However, MD showed more activity to induce apoptotic cell death on PC-9 and C4-2B cancer cells and at 24 h post treatment (Figure 9). To corroborate these results, we analyzed the morphologic change in nuclear condensation and fragmentation present in apoptotic cells. The results showed that MD (100 μg/mL) or MD-6 (50 μM) increased the number of cells with nuclear condensation and nuclear fragmentation at 24 h post treatment. Thus, these results strongly suggest that MD and MD-6 induce apoptosis in breast, lung, and prostate cancer cell lines (Figure 10).

## 4. Discussion

The study of natural products with cytotoxic activities against cancer cells is an important aim in medicinal chemistry. According to this aim, our research is focused on the isolation of bioactive compounds from the Chilean flora and the understanding of the activity of these molecules in cells. In this study, three compounds are reported for the first time in *Maytenus disticha*; one tricycle sesquiterpene, MD-6 (70 mg, colorless crystals, 0.001% yield), together with two flavonoids, MD-9 (150 mg, white solid, 0.0021% yield) and MD-10 (480 mg, white solid, 0.0069% yield), and these compounds could be used as phytomarkers for *Maytenus disticha*. All NMR spectra are shown in the supplementary information with an excellent agreement to the previously reported structures [31,32]. The MD-6 is a β-dihydroagarofuran sesquiterpene previously isolated from *Schaefferia argentinensis* [33], meanwhile MD-9 is a flavanonol called ampelopsin or dihydromyricetin which displays a broad range of activities, such as inhibitory effects on angiogenesis [34], hepatoprotector [23], anti-inflammatory [35,36], and anti-alcohol intoxication [37] and MD-10 a 3-O-β-glycoside of dihydromyricetin with no present reports on its activity. The extract of leaves and seeds of *M. disticha* have been previously studied, discovering only β-dihydroagarofuran sesquiterpenes as the main constituents with AChE inhibitory activity [11]. In this research, we showed that MD has cytotoxic activity against human cancer cells, inducing mitochondrial membrane depolarization and increasing in mitochondrial superoxide generation, which induces apoptosis. Supporting these results, other groups reported the pro-apoptotic activity of plants of the genus *Maytenus*. In this sense, total extracts of *Maytenus royleanus* showed potent antiproliferative and pro-apoptotic effects in human prostate cancer cells promoting the activation of Caspase-3 and PARP cleavage [38]. Moreover, a dry *Maytenus ilicifolia* extract protects normal cells and induces apoptosis, decreasing BCL-2 in human cancer cells [39]. Furthermore, we demonstrate that the cytotoxic activity of MD could be induced in part by MD-6, a β-dihydroagarofuran sesquiterpene, which shows a different structure compared with the compounds previously isolated [40], for instance, an opposite configuration of the carbon 9 and different oxidation at position C4. We observe that MD6 induces a mitochondrial superoxide and general cellular ROS generation. In this sense, it has been described that the release of cytochrome C from the mitochondria, a key step in the apoptosis, is facilitated by ROS [41]. Actually, ROS generation produces cytochrome C releasing by two processes: the generation of mitochondrial pores formed by BAX/BAK proteins and the induction of mitochondrial permeability transition (MPT) [42]. Consistent with this evidence, we demonstrated that MD increases the BAX expression, promotes a mitochondrial membrane potential depolarization, and induces apoptosis in cancer cells. Altogether, our results suggest that MD induces apoptosis by the generation of mitochondrial oxidative stress and mitochondrial dysfunction but not by general oxidative stress generation, because MD only increases mitochondrial superoxide, but does not induce total cellular ROS generation. The paradoxical effects in general ROS generation in MD total extracts, compared with the activity promoted by MD6, could be explained by the presence of flavonoids, such as MD-9 and MD-10, which are important antioxidants [35,36] and counteract the cellular ROS generation.

The effects on the mitochondrial function of other dihydroagarofuran-type sesquiterpenes have been reported. For example, dihydroagarofuran-type sesquiterpenes polyesters have inhibitor properties in the mitochondrial function by binding to the subunit H of the protein V-ATPase, which affects the digestive system of insects [16,17]. Furthermore, some sesquiterpenoids showed trypanocidal activity by mitochondrial dysfunction and ROS generation, for instance, the sesquiterpenoids isolated from *Maytenus boaria* generate cytosolic vacuolization, autophagic phenotype, and mitochondrial swelling [16,17]. In conclusion, dihydroagarofuran-type sesquiterpenes, as the ones isolated in the present research, are important natural molecules present in plants of the *Maytenus* genus, and could be utilized as molecular scaffolds for the development of new anticancer agents that target apoptosis promotion by mitochondrial dysfunction.

## Supplementary Materials

The following are available online at https://www.mdpi.com/2218-273X/10/3/377/s1, Table S1: 1H (600 MHz) and 13C (150 MHz) NMR Data for Isolated Compounds From *Maytenus disticha* in Acetone-d6, Chemical Shifts (δ) in ppm, Coupling Constants (*J*) in Hz, Table S2: Crystal Data, Details of Intensity Measurements, and Structure Refinement for MD-6, Figure S1: ORTEP Plot (50% Probability Ellipsoids) of MD-6, Figure S2: Packing Diagram of MD-6. Hydrogen Bonds as Dashed Lines. For Symmetry Operators—Table S3, Table S3: Hydrogen-Bonding Parameters [Å, °] for MD-6, Table S4: Selected Bond Lengths [Å] for MD-6, Table S5: Selected Bond Angles [°] for MD-6, Figure S3: 1H NMR spectra of MD-6, 600 MHz, in CDCl_3_, Figure S4: 13C NMR spectra of MD-6, 150 MHz, in CDCl_3_, Figure S5: g.s. HSQC spectra of MD-6, in CDCl_3_, Figure S6: g.s. HMBC spectra of MD-6, in CDCl_3_, Figure S7: 1H NMR spectra of MD-9, 600 MHz, in acetone-d6, Figure S8: 13C NMR spectra of MD-9, 150 MHz, in acetone-d6, Figure S9: g.s. HSQC spectra of MD-9, in acetone-d6, Figure S10: g.s. HMBC spectra of MD-9, in acetone-d6, Figure S11: 1H NMR spectra of MD-10, 600 MHz, in acetone-d6, Figure S12: 13C NMR spectra of MD-10, 150 MHz, in acetone-d6, Figure S13: g.s. HSQC spectra of MD-10, in acetone-d6, Figure S14: g.s. HMBC spectra of MD-10, in acetone-d6.
