# Supplementary material for: Maytenus disticha Extract and an Isolated β-Dihydroagarofuran Induce Mitochondrial Depolarization and Apoptosis in Human Cancer Cells by Increasing Mitochondrial Reactive Oxygen Species
Source: Biomolecules. 2020 Feb 29;10(3):377. doi: 10.3390/biom10030377 (PMC7175306; doi:10.3390/biom10030377)

Supplementary Material

Maytenus disticha Extract and an Isolated β-Dihydroagarofuran Induce Mitochondrial Depolarization and Apoptosis in Human Cancer Cells by Increasing Mitochondrial Reactive Oxygen Species

Iván González-Chavarría [1], Felix Duprat [1], Francisco Roa[1], Nery Jara[5], Jorge Toledo [1], Felipe Miranda [2], José Becerra [3], Alejandro Inostroza [3], Alexandra Kelling [4], Uwe Schilde [4], Matthias Heydenreich [4] and Cristian Paz [2*]

Table S1. ^{1}H (600 MHz) and ^{13}C (150 MHz) NMR Data for Isolated Compounds From *Maytenus disticha* in Acetone-d6. Chemical Shifts (δ) in ppm, Coupling Constants (*J*) in Hz.

	**^{1}H NMR**			**^{13}C NMR**		
	MD-6[a]	MD-9	MD-10	MD-6[a]	MD-9	MD-10
**1**	5.40, dd, 12.4, 4.0	-	-	76.2	-	-
**2**	1.81, m<br>1.45, m	4.95, d, 11.3	5.03, d, 11.4	25.2	84.5	84.1
**3**	1.94, td, 14.4, 4.6<br>1.81, m	4.56, dd, 11.3, 2.3	4.61,d, 11.4	38.2	73.2	73.1
**4**	-	-	-	70.4	198.1	197.9
**5**	-	-	-	93.7	165.0	165.0
**6**	6.64, d, 0.8	5.97, d, 2.1	5.99, d, 2.1	74.4	97.0	97.1
**7**	2.98, d, 0.8	-	-	65.1	167.8	167.8
**8**	-	5.94, d, 2.1	5.97, d, 2.1	197.8	96.0	96.1
**9**	5.90, s	-	-	79.6	164.2	163.9
10	-	-	-	52.0	100.0	101.5
11	-	-	-	84.9	129.0	136.9
12	1.64, s	6.62, 2H, s	6.66, 2H, s	29.4	108.0	108.0
13	1.61, s	-	-	24.7	146.3	151.0
14	1.39, s	-	-	23.8	134.2	134.7
15	5.09, d, 12.7<br>4.32, d, 12.7		-	60.6	-	-
1'	-	-	5.03, d, 11.4	-	-	84.1

[a]: in $CDCl_3$

Table S2. Crystal Data, Details of Intensity Measurements, and Structure Refinement for MD-6

Chemical formula	$C_{28}H_{34}O_{11}$
Molecular weight	546.55
Crystal system	orthorhombic
Space group	$P2_12_12_1$
a / Å	8.8254(2)
b / Å	11.4351(2)
c / Å	26.7639(7)
Unit cell volume / $Å^3$	2701.0(1)
Temperature / K	210
Z	4
Density (calculated) / $g{\cdot}cm^{-1}$	1.344
Radiation type	MoK_{α}
μ / mm^{-1}	0.104
Reflections collected	93176
Independent reflections	4748
R_{int}	0.0324
*R*1 / *wR*2 [$I > 2\sigma(I)$]	0.0287 / 0.0807
*R*1 / *wR*2 (all data)	0.0300 / 0.0814
Goodness of fit on F^2	1.040

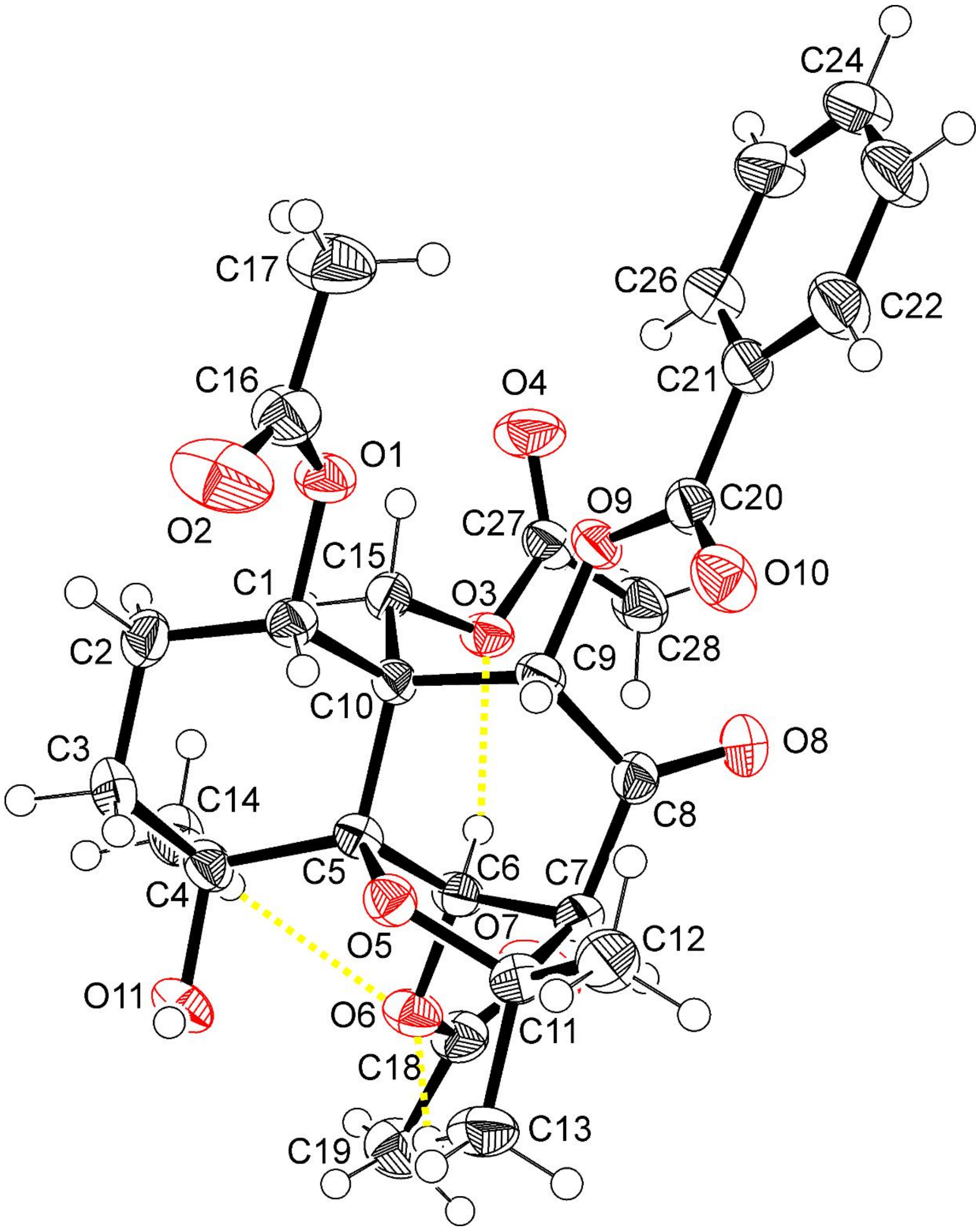

Figure S1. ORTEP Plot (50 % Probability Ellipsoids) of MD-6.

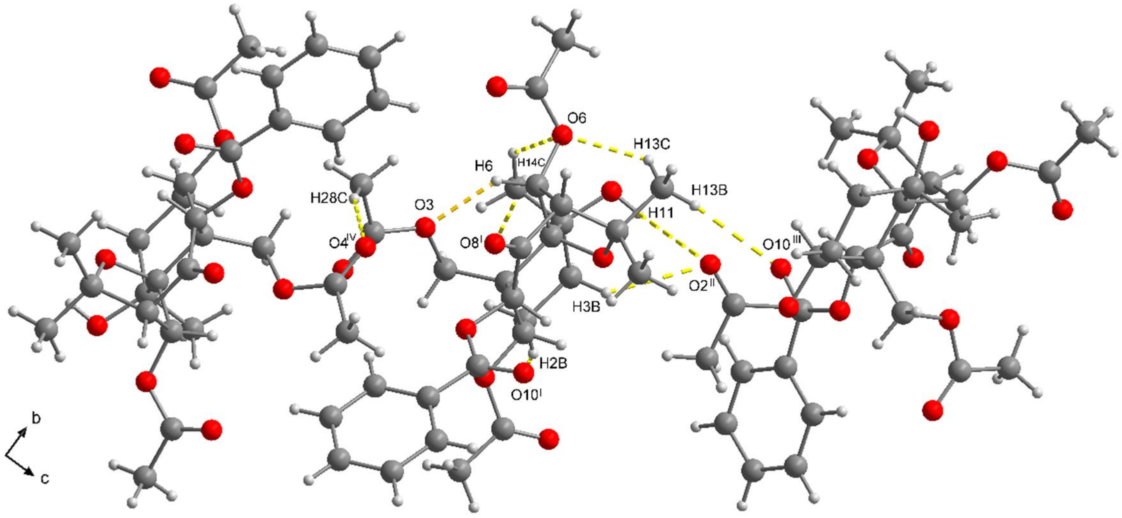

Figure S2. Packing Diagram of MD-6. Hydrogen Bonds as Dashed Lines. For Symmetry Operators – see Table S3.

Table S3. Hydrogen-Bonding Parameters [Å, °] for MD-6.

	D – H	H ··· A	D ··· A	D – H ··· A
C2 – H2B ··· O10^{I}	0.98	2.64	3.355(3)	129.9
C3 – H3B ··· O2^{II}	0.98	2.65	3.400(3)	133.1
C6 – H6 ··· O3	0.99	2.06	2.888(2)	140.0
C13 – 13B ··· O10^{III}	0.97	2.64	3.481(3)	145.3
C13 – H13C ··· O6	0.97	2.23	2.948(3)	130.3
C14 – H14B ··· O6	0.97	2.44	3.071(3)	122.8
C14 – H14C ··· O8^{I}	0.97	2.63	3.284(3)	124.8
C28 – H28C ··· O4^{IV}	0.97	2.65	3.585(3)	162.8
O11 – H11 ··· O2^{II}	0.83(3)	2.33(3)	3.101(2)	154(3)

Symmetry operators: ^{I} 1+x,y,z; ^{II} 2-x,0.5+y,0.5-z; ^{III} 1–x,0.5+y,0.5-z; ^{IV} x-0.5,-y-0.5,-z

Table S4. Selected Bond Lengths [Å] for MD-6.

C16 – O2	1.199(3)
C27 – O4	1.190(3)
C6 – O6	1.439(2)
C8 – O8	1.202(3)
C20 – O10	1.204(3)
C14 – O11	1.429(2)
O11 – H11	0.83(3)

Table S5. Selected Bond Angles [°] for MD-6.

C1 – O1 – C16	116.2(2)
O1 – C16 – O2	123.4(2)
C15 – O3 – C27	118.5(2)
O3 – C27 – O4	124.0(2)
C3 – C4 – O11	107.6(2)
C5 – O5 – O11	111.1(1)
C5 – C6 – O6	112.9(2)
C6 – O6 – C18	116.0(2)
O6 – C18 – O7	122.9(2)
C7– C8 – O8	123.5(2)
C9 – O9 – C20	114.6(2)
C9 – C20 – O10	123.3(2)

Figure S3. ^{1}H NMR spectra of MD-6, 600 MHz, in $CDCl_3$.

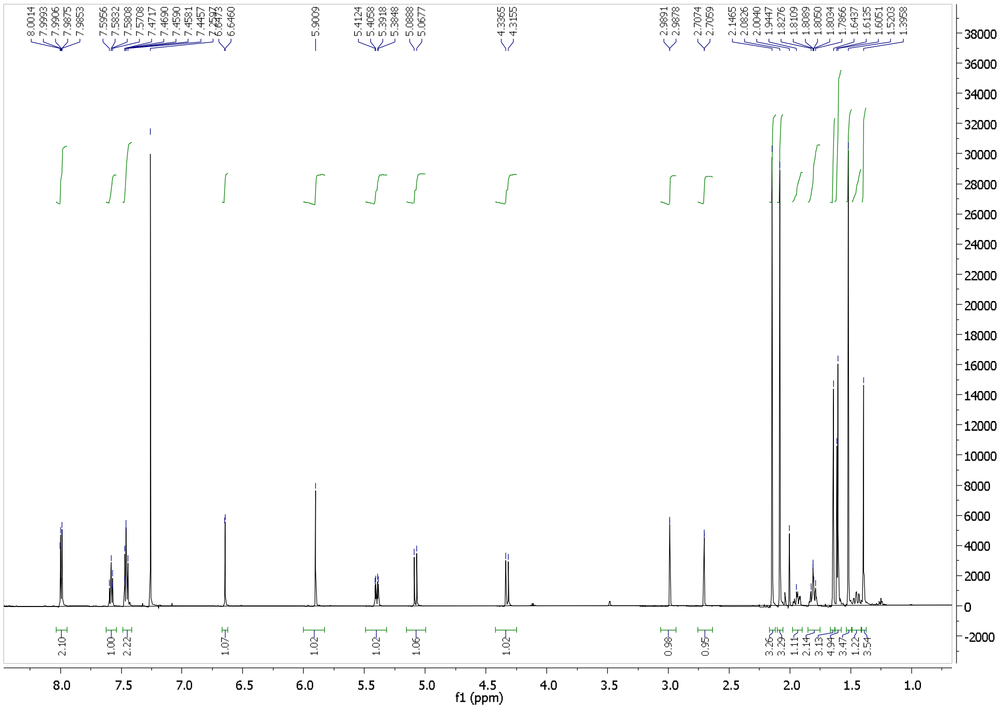

Figure S4. ^{13}C NMR spectra of MD-6, 150 MHz, in $CDCl_3$.

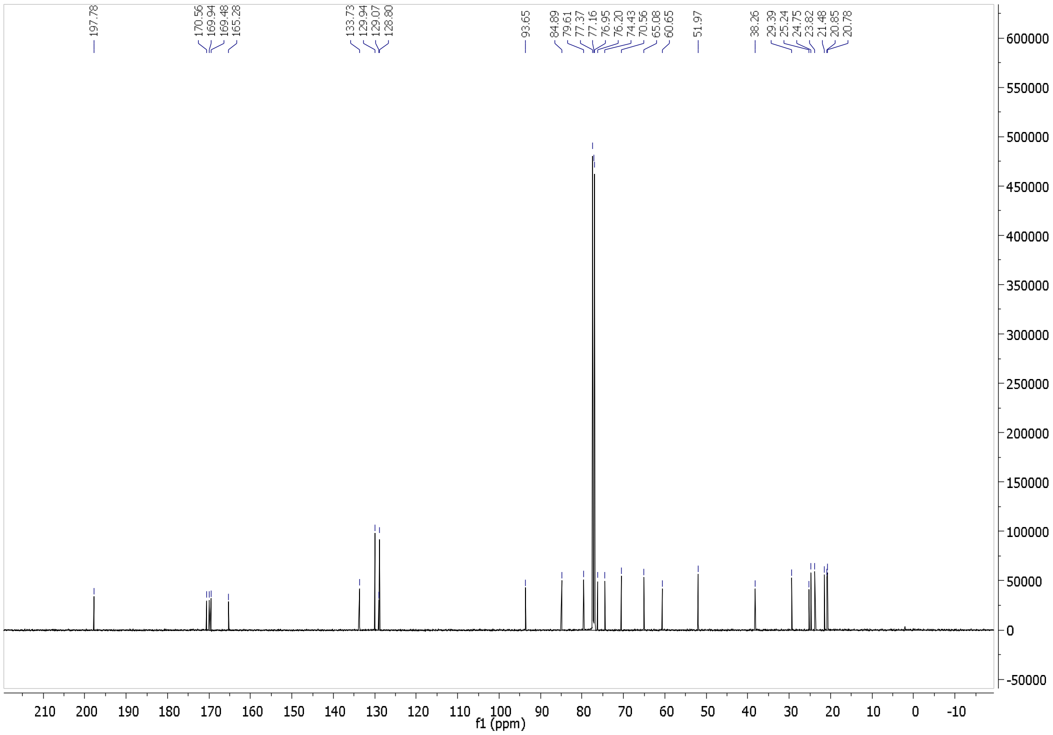

Figure S5. g.s. HSQC spectra of MD-6, in $CDCl_3$.

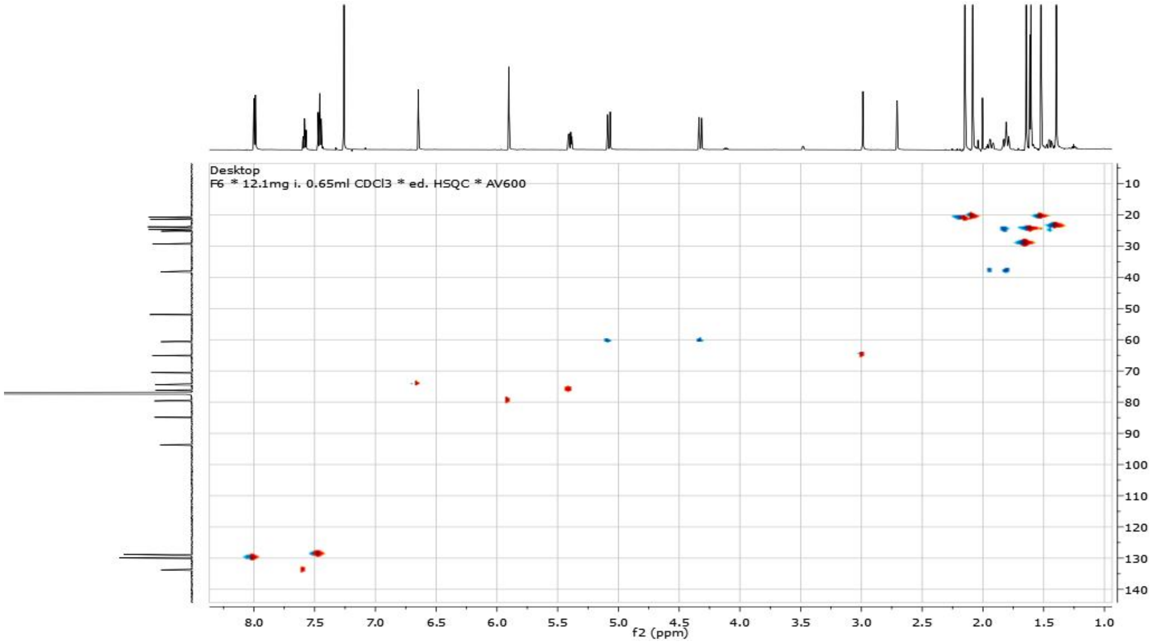

Figure S6. g.s. HMBC spectra of MD-6, in $CDCl_3$.

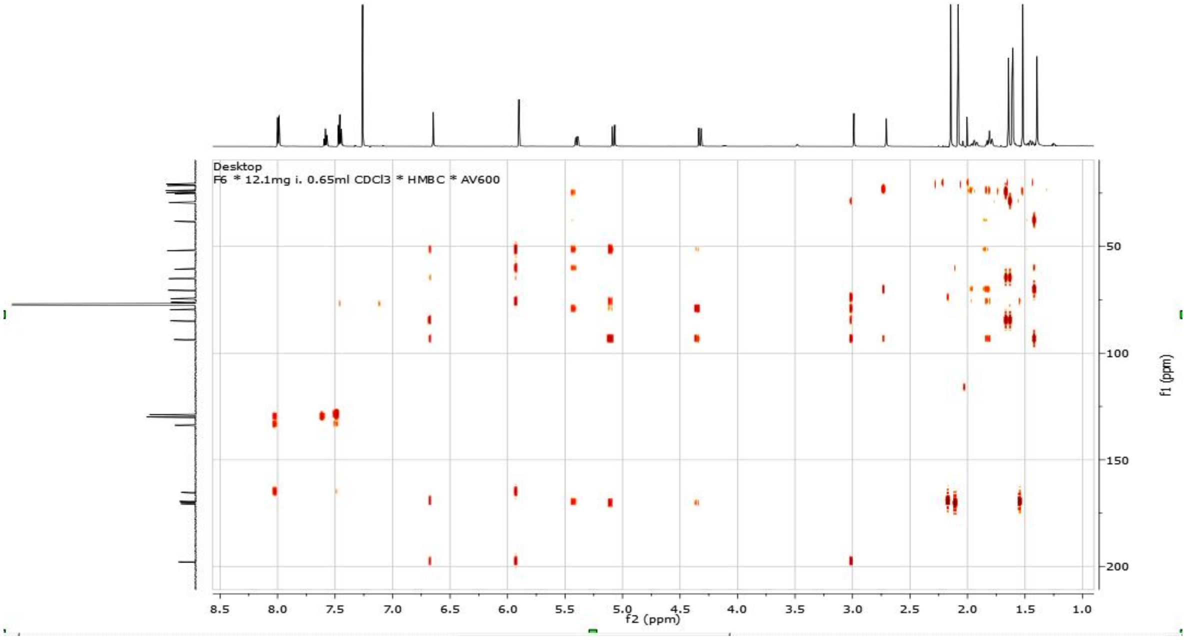

Figure S7. ^{1}H NMR spectra of MD-9, 600 MHz, in acetone-d6.

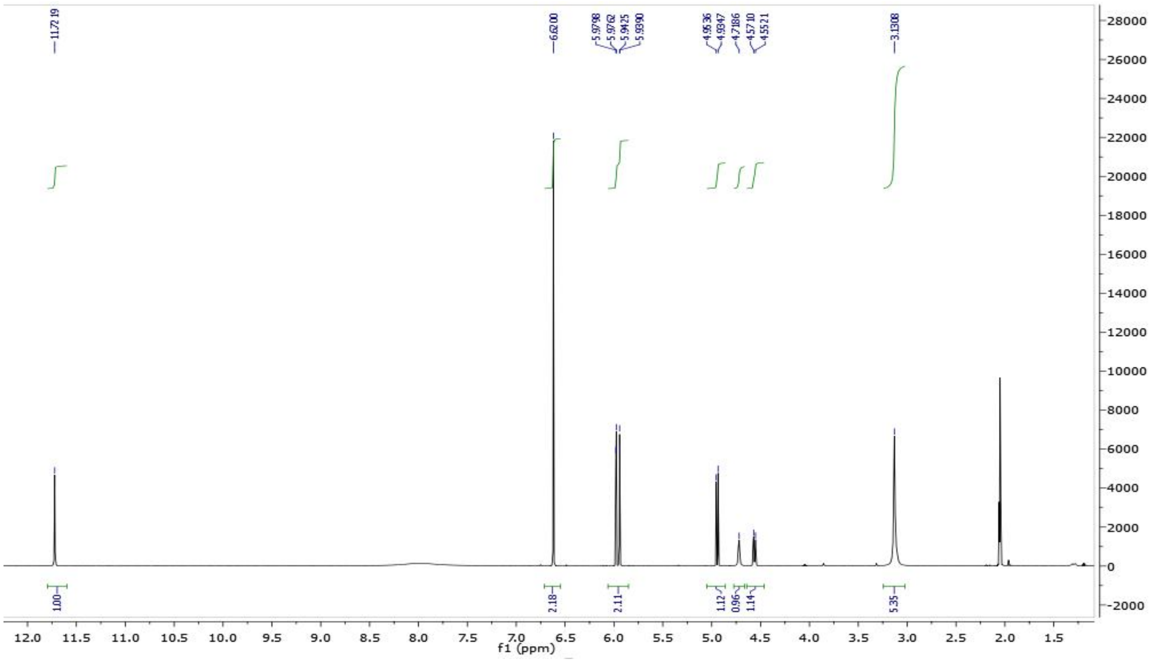

Figure S8. ^{13}C NMR spectra of MD-9, 150 MHz, in acetone-d6.

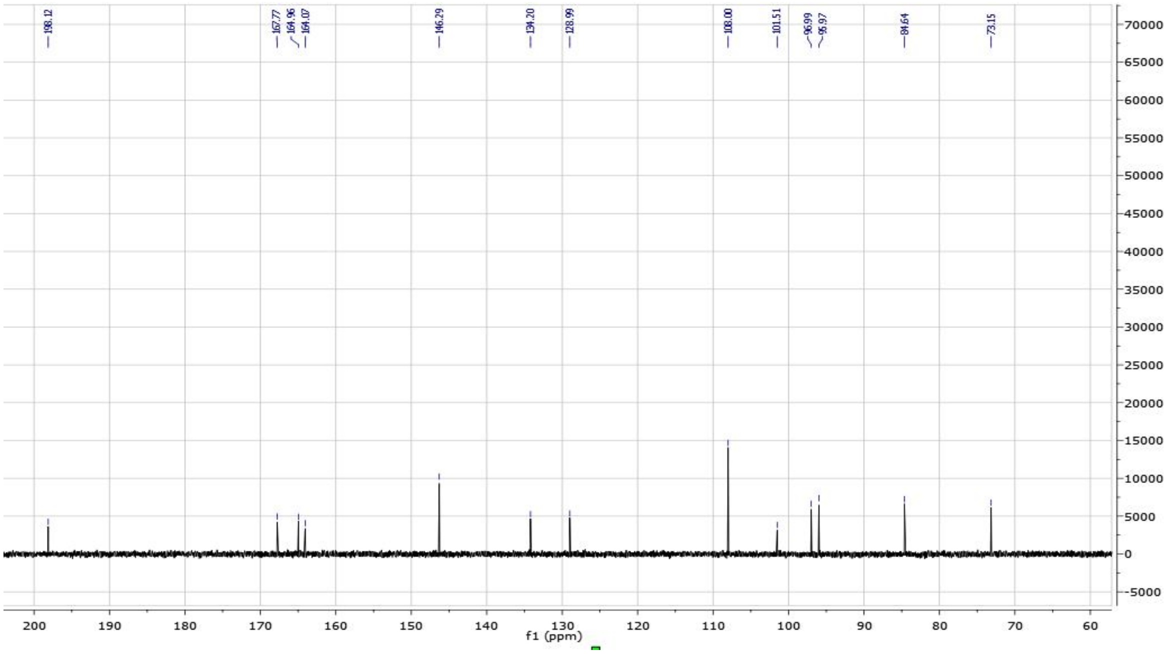

Figure S9. g.s. HSQC spectra of MD-9, in acetone-d6.

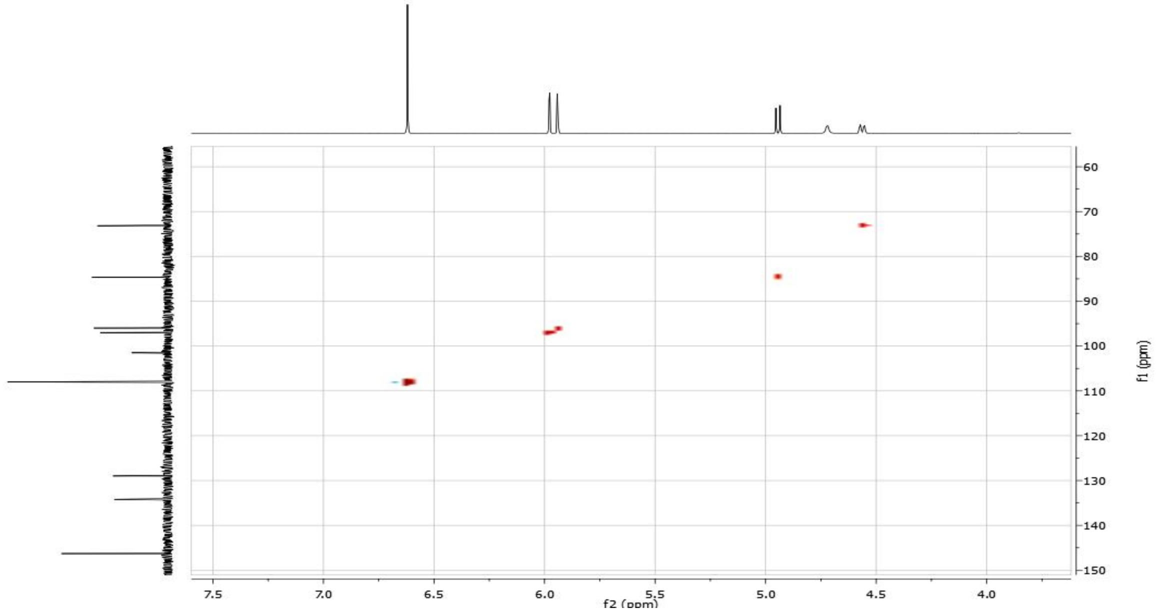

Figure S10. g.s. HMBC spectra of MD-9, in acetone-d6.

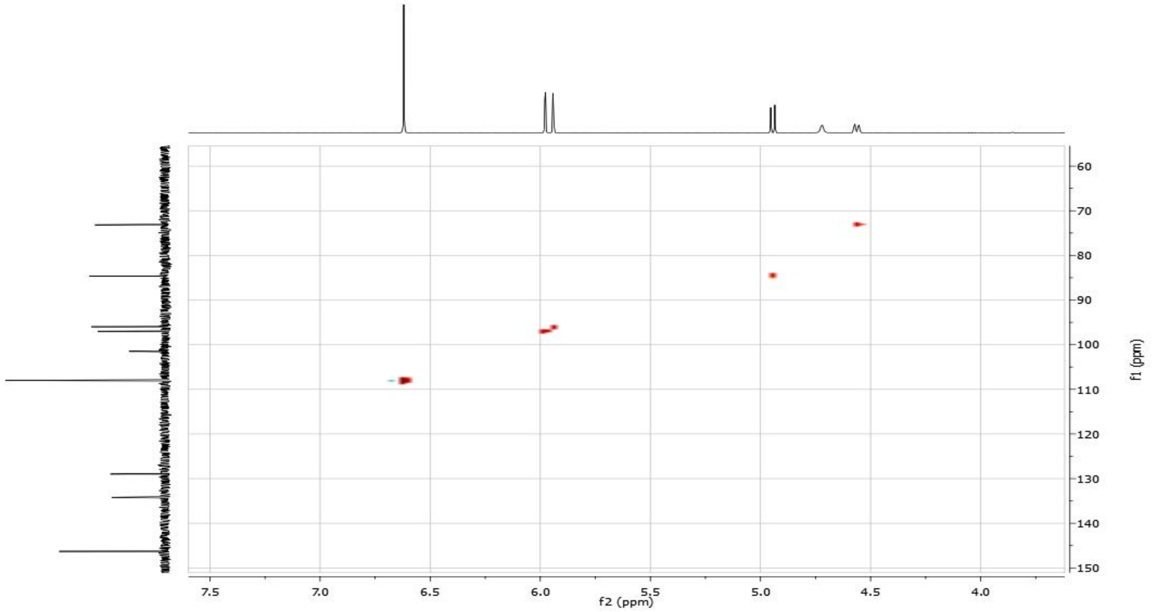

Figure S11. ^{1}H NMR spectra of MD-10, 600 MHz, in acetone-d6.

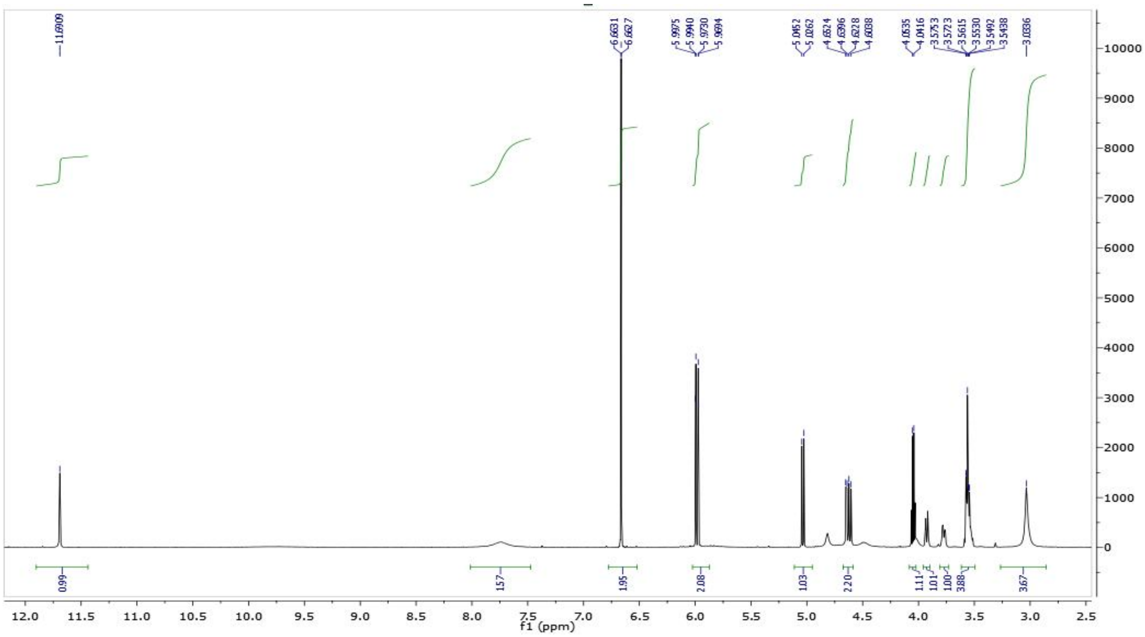

Figure S12. ^{13}C NMR spectra of MD-10, 150 MHz, in acetone-d6.

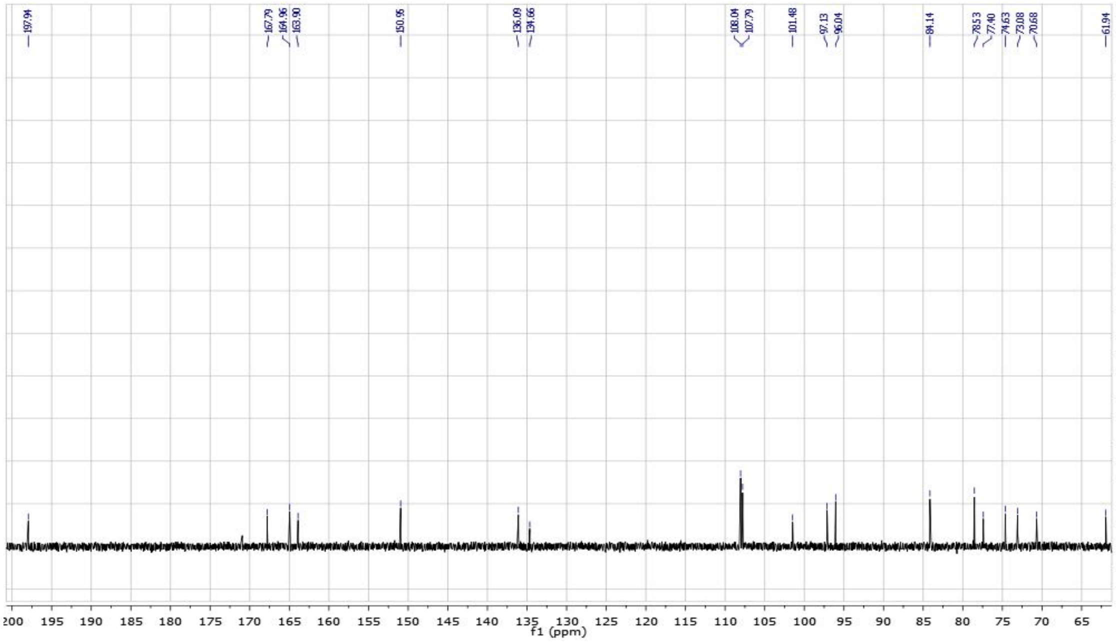

Figure S13. g.s. HSQC spectra of MD-10, in acetone-d6.

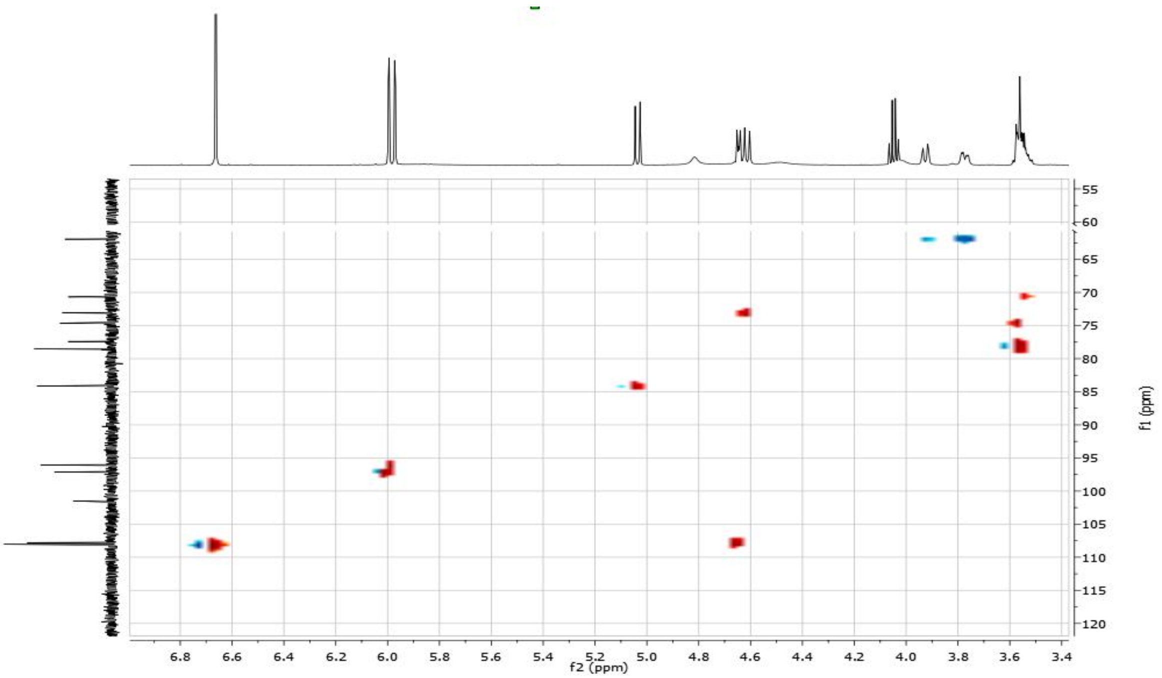

Figure S14. g.s. HMBC spectra of MD-10, in acetone-d6.

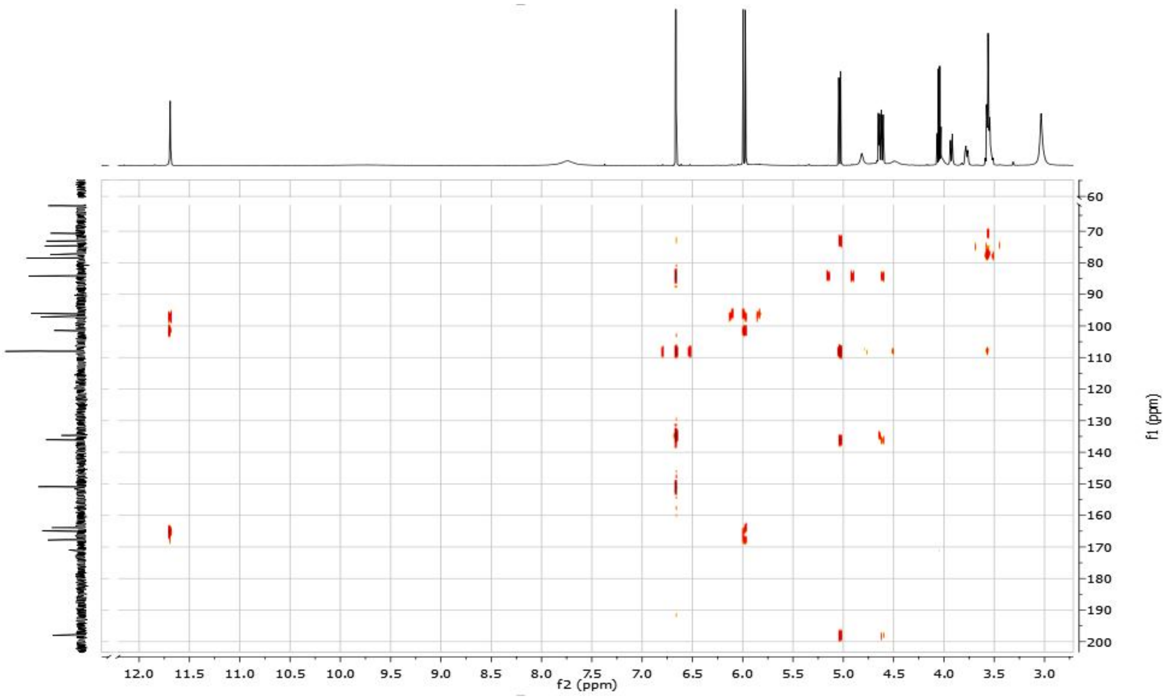

Supplement: Supplementary file 1 [file biomolecules-10-00377-s001.pdf]
